# Outpatient Otolaryngology in the Era of COVID-19: A Data-Driven Analysis of Practice Patterns

**DOI:** 10.1177/0194599820928987

**Published:** 2020-05-12

**Authors:** David A. Kasle, Sina J. Torabi, Emily L. Savoca, Benjamin L. Judson, R. Peter Manes

**Affiliations:** 1 Division of Otolaryngology, School of Medicine, Yale University, New Haven, Connecticut, USA

**Keywords:** COVID-19, telehealth, finances, otolaryngology, health care

## Abstract

**Introduction:**

Coronavirus disease 2019 (COVID-19) has induced a prioritization of acute care and telehealth, affecting the quantity of patients seen and the modality of their care.

**Study Design:**

Retrospective review.

**Setting:**

Single-institution study conducted within the Division of Otolaryngology at the Yale School of Medicine.

**Subjects and Methods:**

Data on all outpatient appointments within the Division of Otolaryngology were obtained from administrative records of billing and scheduling from March 16 to April 10, 2020. For comparison, a corresponding period from 2019 was also utilized.

**Results:**

Of 5913 scheduled visits, 3665 (62.0%) were seen between March 18 and April 12, 2019, in comparison with 649 of 5044 (12.9%) during the corresponding COVID-19–affected period. The majority of completed visits performed in weeks 1 and 2 were in person, while the majority in weeks 3 and 4 were via telehealth. Among subspecialties, a larger proportion of completed visits in 2020 were performed by pediatric and head and neck oncology otolaryngologists as compared with general/specialty otolaryngologists (*P* < .001). Older adults (≥65 years) were less likely to have telehealth visits than younger adults (18-64 years; 45.6% vs 59.6%, *P* = .003).

**Conclusions:**

A major decrease in the completion rates of scheduled visits was seen in the COVID-19–affected period, though this was not proportional among subspecialties. An associated increase in telehealth visits was observed. After COVID-19–related hospital policy changes, approximately 2 weeks passed before telehealth visits surpassed in-person visits, though this was not true among older adults.

Coronavirus disease 2019 (COVID-19), the illness caused by a novel coronavirus identified in December 2019, was first observed in clusters of patients in Wuhan, Hubei Province, China.^1,2^ The World Health Organization declared COVID-19 to be a public health emergency of international concern in January 2020 and ultimately a pandemic in March 2020.^2,3^ The virus’s particularly high rate of transmission prompted implementation of various widespread restrictions, including bans on international travel and government-mandated lockdowns, in an attempt to contain its spread.^4^ Nonetheless, by March 2020, well over 50,000 people in 25 countries had confirmed cases of COVID-19, and patients with severe respiratory symptoms were associated with a higher rate of fatality.^5^

As health care systems grapple with increasing numbers of patients, attempts to protect patients and providers, in addition to preserving personal protective equipment, have elicited a necessary triaging of care. This has taken form in rescheduling nonacute appointments and utilizing telehealth programs.^6^ Otolaryngologists may be particularly at risk due to a high viral load in the upper aerodigestive tract and the risk of aerosolization inherent to common otolaryngologic procedures. In light of this, there has been a widespread shift among otolaryngologists toward minimizing direct patient interaction in nonacute settings.^7,8^ Several otolaryngology divisions and departments have documented their subjective response to this changing landscape, but little objective measurement on COVID-19’s impact has been reported.^9,10^

Herein, we discuss the affect that COVID-19 has had on our institution. The primary objectives were to examine the quantitative change in patient visits, as well as the modality in which they were completed, over the first month of the virus’s impact within the United States, in comparison with the prior year. Secondarily, we analyzed trends in weekly visitations, differences among otolaryngology subspecialties, and disparities among patients of varying ages.

## Methods

### Setting

This was a single-institution study conducted within the entirety of the Division of Otolaryngology at the Yale School of Medicine. This is an academic tertiary care center but also includes community clinics. The geographic area of our clinics and patients is throughout southern Connecticut. This study was exempted from review by the Yale Human Investigation Committee due to secondary research on existing data sets.

### Period and Data

As the focus of study was on COVID-19’s impact on otolaryngology services, we queried the first 4 weeks of the crisis during which departmental functions were affected—from March 16 to April 10, 2020 (henceforth, the 2020 period). For a comparison group, we queried the corresponding period from the previous year—March 18 to April 12, 2019 (henceforth, the 2019 period). Data on all outpatient appointment within the otolaryngology division were obtained from administrative records of billing and scheduling.

### Variables

The data consisted of the following variables: date of scheduled appointment, completion status of that appointment, reason for cancellation (if it was canceled), type of appointment (in person or telehealth), provider name, and patient age.

Dates were grouped into weeks 1 to 4 for each period. Appointment completion status was grouped into completed, cancelled, no-show, or left without being seen. If the appointment was cancelled, cancellation reasons were grouped into COVID-19 and non–COVID-19 administrative-directed cancellations or patient-directed cancellations. To qualify as a COVID-19 administrative-directed cancellation, the appointment must have been in 2020 and must have a cancellation reason of “crisis planning” or “bump.” No instances of the former reason existed in 2019, though 12 of 108 (11.1%) bumps occurred in 2019 (which were categorized as non–COVID-19 administrative-directed cancellations). “Video” or “telephone” visits were determined to be telehealth visits, and all other visit types were grouped as in person.

Provider name was utilized to categorize appointments by subspecialty: pediatric otolaryngology, head and neck oncology, general/specialty otolaryngology (which included general otolaryngology, otologists, laryngologists, rhinologists, and facial plastic surgeons), speech and language pathology (SLP), audiology, and advanced practice providers (nurse practitioners and physician assistants). Physician categorization was determined a priori and based on hospital service workflow and institutional clinic distribution at our academic center. Patients were grouped into functional age cohorts: ≤2, 3-10, 11-17, 18-44, 45-64, 65-79, and ≥80 years.

### Statistical Analysis

Descriptive statistics are presented for scheduled appointments within each period, characterizing the number of completed and cancelled visits overall and stratified by week, specialty, and age. We also provide descriptive statistics analyzing the evolution in use of telehealth overall, as stratified by subspecialty and age. Chi-square tests were performed to analyze the differences in appointment completion rates between 2019 in-person and 2020 telehealth appointments, between proportions of completed visits of the 3 groups of otolaryngology–head and neck surgery physicians in 2019 and 2020, and telehealth utilization between younger (18-64) and older (≥65) adults for completed visits in 2020.

All figures were created in GraphPad Prism 7 (GraphPad Software). Statistical analysis was performed via SPSS 25.0 (IBM Corp), and significance was set at *P* < .05.

## Results

### Outcome and Setting of Scheduled Visits

**Figure 1.**
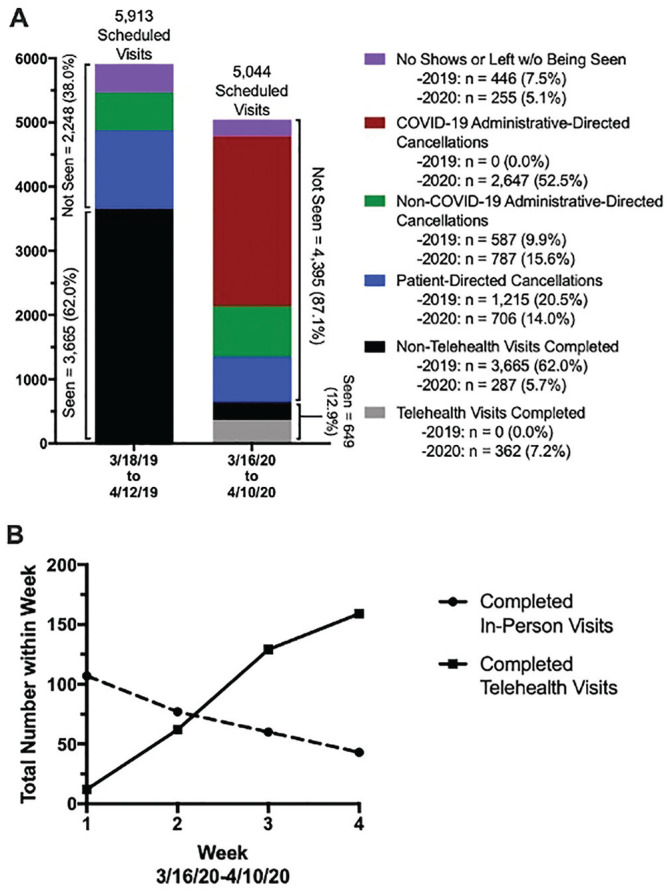
(A) Total breakdown of scheduled visits to the Yale otolaryngology division in 2019 and 2020. (B) Weekly breakdown of number of completed in-person and telehealth visits in 2020.

Between March 18 and April 12, 2019, 5913 visits were scheduled within the otolaryngology division at the Yale School of Medicine. This was distributed to 38 providers: 10 general/specialty otolaryngologists, 7 head and neck oncologists, 3 pediatric otolaryngologists, 3 speech and language pathologists, 10 audiologists, and 5 advanced practice providers. Of these, 3665 (62.0%) visits were completed. In the corresponding COVID-19–affected period (March 16–April 10, 2020), 5044 visits were scheduled, of which 649 were completed (12.9%). More than half of the scheduled visits (n = 2647, 52.5%) in the 2020 period were cancelled due to COVID-19 reasons (**Figure 1A**).

Of the 649 completed visits in the 2020 period, 55.8% were seen via telehealth (**Figure 1A**). When broken down by weeks 1 to 4, the majority of visits performed in weeks 1 and 2 were in person, while the majority in weeks 3 and 4 were via telehealth (**Figure 1B**).

### Telehealth Utilization and Completion Rates

**Figure 2.**
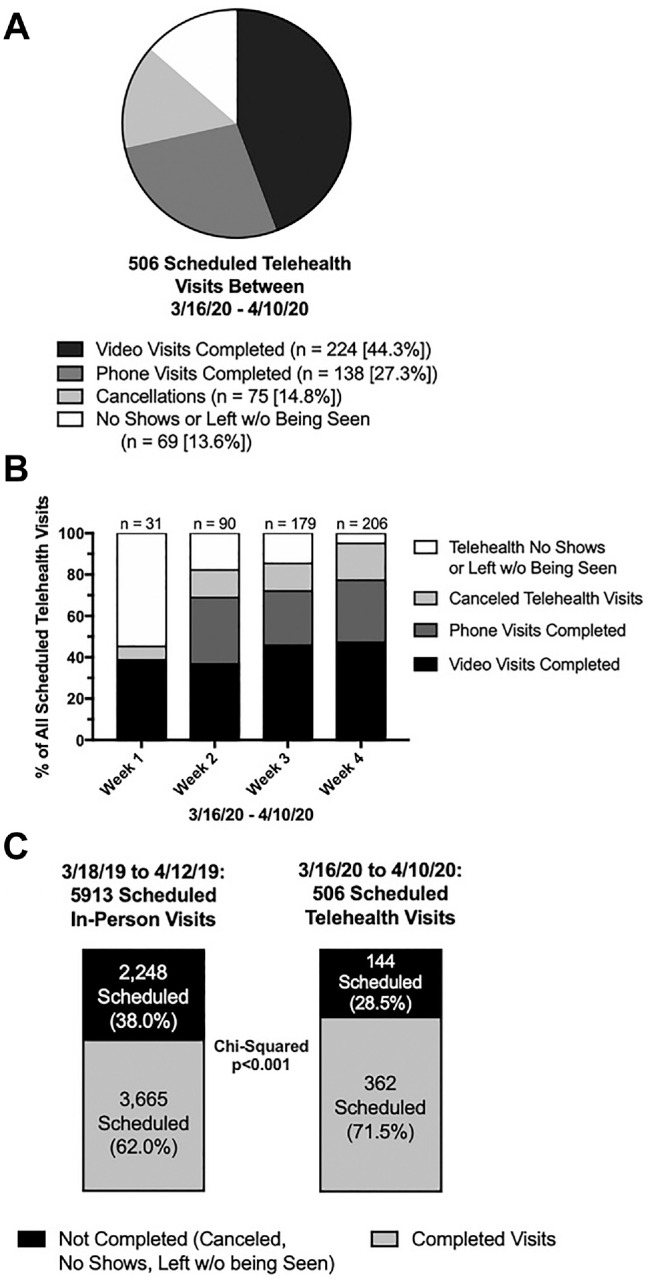
(A) Breakdown of scheduled 2020 telehealth visits. (B) Weekly breakdown of scheduled 2020 telehealth visits. (C) Completion rates of in-person 2019 visits vs telehealth 2020 visits.

Between March 16 and April 10, 2020, there were 506 scheduled telehealth visits. Of these, 362 (71.5%) were completed, of which 224 (61.9%) and 138 (38.1%) were performed via video and telephone, respectively. The overall cancellation and “no-show/left without being seen” rates were roughly equal (14.8% and 13.6%; **Figure 2A**).

When analyzed by week, there was a steep rise in telehealth utilization between weeks 1 and 2 (31 vs 90 scheduled visits) and between weeks 2 and 3 (90 vs 179). Week 4 had the most scheduled telehealth visits, at 206. In week 1, 38.7% of scheduled telehealth visits were completed; however, for the majority of scheduled visits, the patient did not virtually attend or left without being seen (54.8%). The rates of telehealth visit completion rate rose each week; by week 4, only 18.0% of visits were cancelled and 4.9% were categorized as patient no-show/left without being seen (**Figure 2B**). On chi-square analysis, a larger percentage of 2020 scheduled telehealth visits were completed as compared with 2019 scheduled in-person visits (71.5% vs 62.0%, *P* < .001; **Figure 2C**).

### Differences in Subspecialty Practice Patterns During the COVID-19 Pandemic

**Figure 3.**
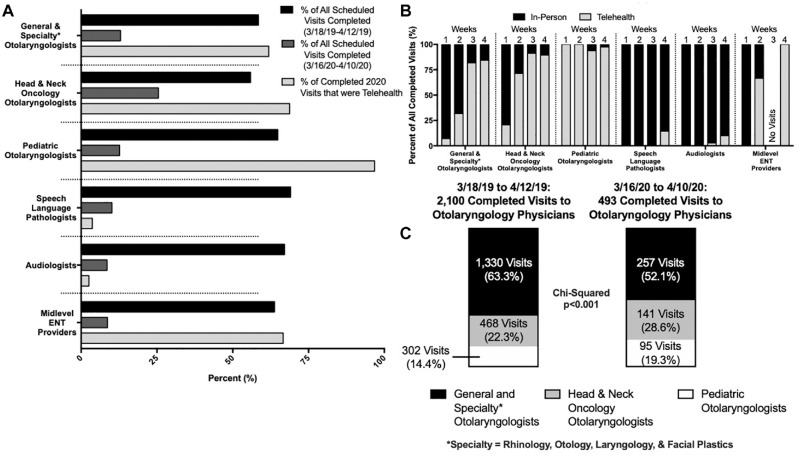
(A) Visits breakdown by specialty. (B) Weekly breakdown of telehealth vs inpatient visits by specialty. (C) Proportion of completed visits to otolaryngology physicians between 2019 and 2020.

During the 2019 period, most subspecialty divisions saw similar appointment completion rates. The lowest rate was seen in head and neck oncology (55.9%), and the highest was seen in SLP (69.1%). During the 2020 period, appointment completion rates fell for all specialties (general/specialty otolaryngology, 13.0%; pediatric otolaryngology, 12.7%; SLP, 10.2%; audiology, 8.6%; midlevel providers, 8.7%) and was highest for head and neck oncology (25.5%). The telehealth utilization rate for completed appointments in 2020 was highest in pediatric otolaryngology (96.8%, 92 of 95 visits). Telehealth utilization rates were 61.8% (159 of 257), 68.8% (97 of 141), and 66.6% (10 of 15) in general/specialty otolaryngology, head and neck oncology, and midlevel providers, respectively. Telehealth utilization was low in audiology (2.6%, 3 of 114) and SLP (3.7%, 1 of 27; **Figure 3A**).

When stratified by week, general/specialty otolaryngology physicians transitioned to a majority telehealth service between weeks 2 and 3, while head and neck oncology physicians and midlevel providers transitioned between weeks 1 and 2. Pediatric otolaryngology providers performed virtually all of their completed appointments over telehealth from the onset, though it is important to note that only 2 patients were seen by this subspecialty during week 1. However, virtually all SLP and audiology visits were completed in person, with a small amount seen via telehealth at the end of the period (**Figure 3B**). Of completed appointments among the 3 major groups of otolaryngology physicians (general/specialty, pediatric, and head and neck oncology), pediatric otolaryngology and head and neck oncology visits each accounted for larger shares of completed visits in 2020 versus 2019, while 2020 general/specialty otolaryngology visits accounted for a smaller share when compared with 2019 (pediatric otolaryngology, 19.3% vs 14.4%; head and neck oncology, 28.6% vs 22.3%; general/specialty otolaryngology, 52.1% vs 63.3%; *P* < .001; **Figure 3C**).

### Patient Age and Practice Patterns During the COVID-19 Pandemic

**Figure 4.**
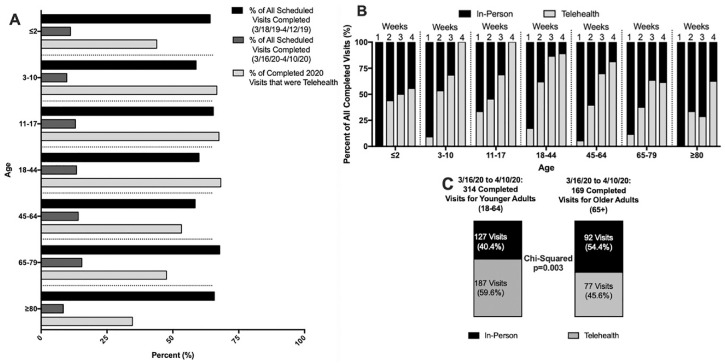
(A) Visits breakdown by age. (B) Weekly breakdown of telehealth vs inpatient visits by age. (C) Proportion of 2020 in-person and telehealth visits between younger (18-64 years) and older (≥65 years) adults.

During the 2019 period, appointment completion rates among the age groups were similar (range, 58.5%-67.8%). During the 2020 period, appointment completion rates significantly decreased across all age groups (≤2 years, 11.1%; 3-10 years, 9.7%; 11-17 years, 13.0%; 18-44 years, 13.4%; 45-64 years, 14.1%; 45-64 years, 14.1%; 65-79 years, 15.4%) and was lowest for the ≥80-year age group, at 8.4%. With the exception of those at the extremes of age (≤2 and ≥65 years), the majority of completed appointments were telehealth visits (range, 53.3%-68.2% for ages 3 to 64 years). The telehealth utilization rate for those ≤2 years was 43.9% (25 of 57); 65 to 79 years, 47.6% (68 of 143); and ≥80 years, 34.6% (9 of 26; **Figure 4A**).

When stratified by week, the telehealth utilization rate of those ≥80 years old lagged behind the other age groups, reaching >50% of visits only during the week 4. The other age groups reached at least 50% during week 2 or 3 (**Figure 4B**). When comparing telehealth utilization between younger (18-64 years) and older (≥65 years) adults in 2020, we saw that 187 (59.6%) of all completed visits for younger adults were via telehealth, which was significantly higher than that of older adults (45.6%, 77 of 169 completed visits; *P* = .003; **Figure 4C**).

## Discussion

Comparable to the drastic adjustment in routine daily living that COVID-19 has necessitated, health care administration has transformed considerably.^11^ Health care systems have revamped their practice patterns in an attempt to conserve personal protective equipment and safeguard patients and providers.^12,13^ This is true among frontline physicians and departments, such as emergency rooms, as well as specialty providers, including otolaryngology.^14-18^ Particularly pertinent to otolaryngology is a heightened concern regarding the increased risk of COVID-19 transmission owing to the nature of the head and neck examination and common otolaryngologic procedures.^7,8^ Furthermore, telehealth and distance care can be especially difficult among subsets of otolaryngology diseases, including head and neck cancers and airway disorders.^19^ As the scope of otolaryngology practice is wide, ranging from benign pathologies (including hearing loss and sinusitis) to head and neck cancer, a shift in the quantity and modality of clinical visits is expected and encouraged. Many institutions have fashioned and employed guidelines regarding triaging patient care conditioned on the acuity of the otolaryngologic condition.^7,8,10^ Nevertheless, with a lack of complete understanding of COVID-19 and its impact, there is expected variability within these recommendations.

At the Yale School of Medicine’s Division of Otolaryngology, routine nonurgent care has been cancelled, rescheduled, or transitioned to telehealth. Patients with features and symptoms concerning for more malignant pathologies are seen in person initially or triaged via telehealth preceding an in-person encounter. This has allowed for fewer direct interactions in the setting of a global pandemic while still providing necessary acute care. In assessing this transition, we analyzed administrative records of our otolaryngology division’s outpatient practice patterns during the first 4 weeks following hospital-wide aggressive COVID-19 policy changes—March 16 to April 10, 2020—and compared them with the corresponding period from 1 year prior. We specifically examined all board-certified otolaryngology–head and neck surgeons, speech and language pathologists, audiologists, and otolaryngologic advanced practice providers (including nurse practitioners and physician assistants).

Of 5044 scheduled encounters, only 649 (12.9%) were completed in the 2020 period, with the majority of visits cancelled secondary to COVID-19, as compared with 62% of 5913 scheduled visits in the 2019 period. Of the 2020 completed visits, 55.8% were via telehealth. In the corresponding 2019 period, no telehealth visits were completed (**Figure 1A**). Few comparable objective data have quantified the changes in outpatient practice to date. One health care technology company, Phreesia, collected and analyzed data from >50,000 physician clients from February 1 through April 16, 2020, utilizing practice management/scheduling software, check-in information provided by patients, and electronic medical records.^20^ Consistent with our findings, the COVID-19–affected period saw a significant decline in outpatient visits from baseline. Interestingly, relative to other ambulatory practices, otolaryngology had the second-greatest decline of in-person visitations—approximately 75%—second only to ophthalmology (79%). As mentioned, concern for increased risk of transmission with close examination of the head and neck may contribute to this and would also apply to ophthalmology. In addition, a relatively large volume of elective, semielective, and nonurgent evaluations likely factors in, especially for the surgical subspecialties.

A weekly increase in telehealth encounters and analogous decrease in in-person encounters were observed among Yale otolaryngology patients, with telehealth surpassing in-person visits at approximately the beginning of the third week (**Figure 1B**). This likely represents a learning curve in implementing and executing telehealth for the institution, provider, and patient. Interestingly, in the first week, almost 55% of scheduled telehealth visits were not completed due to patient no-shows or leaving without being seen; however, by the fourth week, only 18% of visits were not completed for these reasons. Furthermore, by the end of the month, a larger percentage of 2020 scheduled telehealth visits were completed as compared with 2019 scheduled in-person visits (71.5% vs 62.0%, *P* < .001). Patient and institutional comfort and dexterity with a new interactive format likely contributed considerably to this finding (**Figure 2**).

Specifically, in regard to provider type, all subspecialty practices incurred a decrease in appointment completion rates in the 2020 period. However, this drop was lowest among head and neck oncology patients, which may reflect a higher acuity in their pathology. Furthermore, telehealth utilization rates were highest in the pediatric and head and neck oncology subspecialties, though all specialties exhibited a trend of increasing telehealth usage over the course of 1 month (**Figure 3A and **3B****). While the precise reasoning for higher pediatric and head and neck oncology telehealth utilization rates is unclear, it should be noted that the Yale School of Medicine oversees outpatient visits for general and specialty otolaryngology, while Yale New Haven Hospital, a separate organization, manages the head and neck oncology and pediatric otolaryngology clinics. Another explanation may again include the acuity of care necessitating increased efforts to coordinate visits. Within pediatrics, preexisting comfort level with technology and telehealth among younger parents and teenagers may be a contributing factor. Not surprising, the total number of SLP and audiology visits dropped precipitously in 2020 as compared with 2019, and the majority of 2020 visits occurred in person. SLP and audiology represent fields in which patients are commonly referred for less acute pathologies and examinations are more difficult to perform remotely.

General and specialty otolaryngology visits accounted for 63.3% of overall visits in 2019 but only 52% of visits in 2020. Of all physician visits in 2020, 28.6% and 19.3% were seen by head and neck oncology and pediatric otolaryngology, respectively, a 5%-6% relative increase in each (**Figure 3C**). Once more, the precise reasoning for this cannot be ascertained from our data. However, as mentioned previously, the dual managing Yale organizations and difference in patient populations may account for this distinction.

Appointment completion rates fell across all age groups in 2020 by 10% to 15%, with the lowest completion rates in patients ≥80 years old. There was a subsequent rise in telehealth visits universally. However, while the majority of patients’ care transitioned to telehealth, patients aged ≤2 and ≥80 years received mostly in-person care (43.9% and 34.6% seen via telehealth, respectively; **Figure 4**). Both populations represent at-risk patients historically, which is particularly true among the elderly during the COVID-19 pandemic.^21,22^ Thus, the slower transition to telehealth for these patients is a cause for concern and further analysis.

One inherent limitation of the current study is its retrospective nature. Additionally, only a 4-week time frame was utilized, limiting the number of scheduled visits and the analysis included. It is likely that these trends continue to evolve. Last, this was a single institution’s experience, which can limit the globalization of its findings. Nonetheless, quantitative data can allow other institutions to glean useful information on possible transitioning methods and expected practice alterations. Continuing to monitor trends within health care systems during the COVID-19 pandemic is crucial. Consistent findings of increased in-person interaction among at-risk populations, for example, may promote innovative and useful action in the application of safe care. Furthermore, with a broader call for health care reform and universal access to affordable care, efficient use of resources, via patient triaging and telehealth, can have a profound impact beyond the COVID-19 pandemic.^23^

## Conclusion

COVID-19 has had a rapid and global impact on the triaging and administration of health care. The majority of outpatient visits in our otolaryngology division were rescheduled or cancelled in response to this pandemic. We identified a sharp rise in the number of telehealth visits, which became more efficient in their completion rates over time. Importantly, older adults were more likely to be seen in person and less likely to have telehealth visits as compared with younger adults. Further identification of health care trends is vital to promoting safe and efficient care.

## References

[1] Zhu N Zhang D Wang W, et al A novel coronavirus from patients with pneumonia in China, 2019. N Engl J Med. 2020;382(8):727-733. 31978945 10.1056/NEJMoa2001017 PMC7092803

[2] Shultz J Perlin A Saltzman R, et al Pandemic march: COVID-19’s first wave circumnavigates the globe. Disaster Med Public Health Prep. Published online 4 16, 2020. doi:10.1017/dmp.2020.103 PMC7242913 32295665

[3] World Health Organization. Statement on the Second Meeting of the International Health Regulations (2005) Emergency Committee Regarding the Outbreak of Novel Coronavirus (2019-nCoV). World Health Organization; 2020.

[4] Khosrawipour V Lau H Khosrawipour T, et al Failure in initial stage containment of global COVID-19 epicenters. J Med Virol. Published online 4 16, 2020. doi:10.1002/jmv.25883 PMC7262316 32297980

[5] Rothan HA Byrareddy SN. The epidemiology and pathogenesis of coronavirus disease (COVID-19) outbreak. J Autoimmun. 2020;109:102433. 32113704 10.1016/j.jaut.2020.102433 PMC7127067

[6] Hollander JE Carr BG. Virtually perfect? Telemedicine for COVID-19. N Engl J Med. Published online 3 11, 2020. doi:10.1056/NEJMp2003539 32160451

[7] Kowalski LP Sanabria A Ridge JA, et al COVID-19 pandemic: effects and evidence-based recommendations for otolaryngology and head and neck surgery practice. Head Neck. Published online 4 9, 2020. doi:10.1002/hed.26164 PMC7262203 32270581

[8] Givi B Schiff BA Chinn SB, et al Safety recommendations for evaluation and surgery of the head and neck during the COVID-19 pandemic. JAMA Otolaryngol Head Neck Surg. Published online 3 31, 2020. doi:10.1001/jamaoto.2020.0780 32232423

[9] Parikh SR Bly RA Bonilla-Velez J, et al Pediatric otolaryngology divisional and institutional preparatory response at Seattle Children’s Hospital after COVID-19 regional exposure. Otolaryngol Head Neck Surg. Published online 4 14, 2020. doi:194599820919748 10.1177/0194599820919748 32286910

[10] Saibene AM Allevi F Biglioli F, et al Role and management of a head and neck department during the COVID-19 outbreak in Lombardy. Otolaryngol Head Neck Surg. Published online 4 7, 2020. doi:10.1177/0194599820917914 32255735

[11] Centers for Disease Control and Prevention. Implementation of mitigation strategies for communities with local COVID-19 transmission. Accessed March 27, 2020 https://www.cdc.gov/coronavirus/2019-ncov/downloads/community-mitigation-strategy.pdf

[12] Cinar P Kubal T Freifeld A, et al Safety at the time of the COVID-19 pandemic: how to keep our oncology patients and healthcare workers safe. J Natl Compr Canc Netw. Published online 4 15, 2020. doi:10.6004/jnccn.2020.7572 32294617

[13] Adams JG Walls RM. Supporting the health care workforce during the COVID-19 global epidemic. JAMA. Published online 3 12, 2020. doi:10.1001/jama.2020.3972 32163102

[14] Wee LE Fua TP Chua YY, et al Containing COVID-19 in the emergency room: the role of improved case detection and segregation of suspect cases. Acad Emerg Med. Published online 4 12, 2020. doi:10.1111/acem.13984 PMC7262126 32281231

[15] Cao Y Li Q Chen J, et al Hospital emergency management plan during the COVID-19 epidemic. Acad Emerg Med. 2020;27(4):309-311. 32124506 10.1111/acem.13951 PMC7159322

[16] Poonia S Rajasekaran K. Information overload—a method to share updates among frontline staff during the COVID-19 pandemic. Otolaryngol Head Neck Surg. Published online 4 21, 2020. doi:10.1177/0194599820922988 32315261

[17] Saibene AM Allevi F Biglioli F, et al Role and management of a head and neck department during the COVID-19 outbreak in Lombardy. Otolaryngol Head Neck Surg. Published online 4 7, 2020. doi:10.1177/0194599820917914 32255735

[18] Vukkadala N Qian ZJ Holsinger FC, et al COVID-19 and the otolaryngologist—preliminary evidence-based review. Laryngoscope. Published online 3 26, 2020. doi:10.1002/lary.28672 32219846

[19] Prasad A Carey RM Rajasekaran K Head and neck virtual medicine in a pandemic era: lessons from COVID-19. Head Neck. Published online 4 16, 2020. doi:10.1002/hed.26174 PMC7262175 32298018

[20] Mehrotra A Chernew M Linetsky D, et al What impact has COVID-19 had on outpatient visits? To the Point blog. Commonwealth Fund. 4 23, 2020.

[21] Wang W Tang J Wei F Updated understanding of the outbreak of 2019 novel coronavirus (2019-nCoV) in Wuhan, China. J Med Virol. 2020;92(4);441-447. 31994742 10.1002/jmv.25689 PMC7167192

[22] Li Q Guan X Wu P, et al Early transmission dynamics in Wuhan, China, of novel coronavirus-infected pneumonia. N Engl J Med. 2020;382(13):1199-1207. 31995857 10.1056/NEJMoa2001316 PMC7121484

[23] King JS Covid-19 and the need for health care reform. Published online 4 17, 2020 N Engl J Med. doi:10.1056/NEJMp2000821 32302074

